# Insulin‐like growth factor 1 receptor correlates with verbal memory in ILAE type 2 hippocampal sclerosis

**DOI:** 10.1002/epi.70138

**Published:** 2026-02-05

**Authors:** Henrique Cruz, Amauri Batista de Oliveira‐Júnior, André Fernando Garcia Cortez, Ricardo Lutzky Saute, Ricardo Silva Centeno, Joao Norberto Stávale, Mirian Salvadori Bittar Guaranha, Elza Marcia Targas Yacubian, Esper Abrão Cavalheiro, Joao Pereira Leite, Jose Eduardo Peixoto‐Santos

**Affiliations:** ^1^ Discipline of Neuroscience, Department of Neurology and Neurosurgery Escola Paulista de Medicina, UNIFESP São Paulo Brazil; ^2^ Department of Neurosciences and Behavioral Sciences Ribeirao Preto Medical School, USP São Paulo Brazil; ^3^ Discipline of Neurosurgery, Department of Neurology and Neurosurgery Escola Paulista de Medicina, UNIFESP São Paulo Brazil; ^4^ Unipete, Department of Neurology and Neurosurgery Escola Paulista de Medicina, UNIFESP São Paulo Brazil; ^5^ Department of Pathology Escola Paulista de Medicina, UNIFESP São Paulo Brazil

**Keywords:** declarative memory, hippocampal sclerosis type 2, insulin‐like growth factor 1 receptor, temporal lobe epilepsy

## Abstract

**Objective:**

Long‐term memory deficits are often seen in patients with temporal lobe epilepsy (TLE). Recently, studies showed that patients with hippocampal sclerosis (HS) type 2, which presents with severe neuron loss in CA1 only, performed within the normal range. However, up to 30% of HS type 2 cases have memory deficits. As insulin‐like growth factor 1 (IGF‐1) is related to neurogenesis and memory performance, we sought to investigate a possible link between the expression of IGF‐1 receptor (IGF‐1R) and verbal memory performance among patients with HS type 2.

**Methods:**

We selected 21 patients with left‐side TLE and HS type 2. Based on presurgical neuropsychological assessment, we divided the patients into a group with normal long‐term verbal memory (Preserved group, *n* = 15) and another with memory deficit (Deficit group, *n* = 6). To classify the patients, we used performance on the late recall subitems in the Logical Memory test of the Wechsler Memory Scale and late recall in Rey Auditory Verbal Learning Test (RAVLT). Coronal hippocampal sections at the body level were submitted to immunohistochemistry for NeuN and IGF‐1R to evaluate neuron density and IGF‐1R expression, respectively.

**Results:**

Patients with preserved memory had the same clinical characteristics as those with memory deficit. The groups had no difference on the short‐term subitem of Logical Memory or on RAVLT learning. The Deficit group had lower scores on both long‐term memory subitems. Neuron density was also similar among patients in the Preserved and Deficit groups. IGF‐1R expression was significantly higher in the granule cell layer and in CA2 in the Preserved group compared to the Deficit group, and IGF‐1 expression had strong positive correlation with both the learning and long‐term subitems of RAVLT.

**Significance:**

Lower IGF‐1 pathway activity is associated with long‐term memory deficit in patients with HS type 2.

**Key points**

- Up to 70% of patients with left‐side temporal lobe epilepsy and HS type 2 present with preserved long‐term verbal memory.
- Neuron density was similar between HS type 2 patients with preserved memory and those with memory deficit.
- IGF‐1R expression was higher in the granule cell layer of HS type 2 patients with preserved verbal memory compared to those with deficit.
- Late recall scores were correlated with IGF‐1R expression.

## 1 INTRODUCTION

Hippocampal sclerosis (HS) is the most frequent etiology of pharmacoresistant focal epilepsy.^1^ Patients with mesial temporal lobe epilepsy (TLE) and HS often present a history of early childhood precipitating event, followed by a latent period, with recurrent seizures starting during adolescence.^2^ Although development is normal, patients with HS often present a higher frequency of psychiatric comorbidities than the overall population, including anxiety and depression, and most present significant memory deficits.^3^ Neuropsychological assessment of memory and executive functions is mandatory prior to surgical treatment, as the patients must be aware of presurgical status and predict possible postsurgical deficits.

In 2013, the International League Against Epilepsy (ILAE)'s Diagnostic Commission published a consensus classification recognizing three HS types.^4^ Most patients present with HS type 1 or classical HS, with severe neuron loss in CA4/CA3 and CA1, moderate loss in the granule cell layer (GCL), and preservation of CA2 and the subiculum (SUB). Atypical HS include HS type 2, with severe neuron loss only in CA1, and HS type 3, with severe loss in CA4/CA3. The differential subfield loss is linked to the clinical history of the patients, such as childhood febrile seizures and presence of dual pathology, and also predicts surgical outcome.^5^ Memory deficits were also associated with HS types. Whereas patients who present with neuron loss in CA4 (HS types 1 and 3) in the left hemisphere have poor verbal memory, those without HS or with loss confined to CA1 (HS type 2) have preserved verbal memory.^6^, ^7^ Reinforcing these findings, previous studies in HS pointed to the GCL as the most important region to predict memory; granule cell density has a high correlation with memory scores, and patients with reduced granule cell proliferation have worse memory than those with higher proliferative capacity.^8^, ^9^, ^10^ In a previous study, we also reinforced the importance of granule cells to memory in HS type 1, where patients with higher dendritic spines expressing drebrin in the outer molecular layer have better memory score than patients with lower staining in this region.^11^

Besides neuron density, pathways related to cell viability, growth, and proliferation could also impact neuropsychological tests. Among several neurotrophic factors, insulin‐like growth factor 1 (IGF‐1) is implicated in cell growth and differentiation and acts as a antiapoptotic factor.^12^ This factor is so crucial for development that knockout mice have a low birthweight and very short life span, and brain tissue presents several abnormalities.^13^ As IGF‐1 and its receptor (IGF‐1R) are crucial for hippocampal neurogenesis,^14^, ^15^ they may affect hippocampal‐dependent memory. Reinforcing the role of IGF‐1 for memory, a maternal diabetes model indicated a reduction of hippocampal IGF‐1R and memory deficits in the offspring.^16^ IGF‐1R dysregulation was also linked to a neurodegenerative process in animal models, and reduced expression was seen in the brain of patients with Alzheimer disease.^17^ As HS type 2 presents with preservation or, at most, mild neuron loss in the GCL, our hypothesis is that the expression of IGF‐1R in this regions correlates with verbal memory performance in patients with left TLE.

## 2 MATERIALS AND METHODS

### 2.1 Patients

We selected 21 patients with pharmacoresistant left mesial TLE who presented ILAE HS type 2 on neuropathological evaluation among those submitted to surgery at the Epilepsy Treatment Center of our institution. Inclusion criteria were as follows: age between 18 and 60 years, seizure onset in left mesial structures on video‐electroencephalographic evaluation, and neuropsychological workup including memory tests. Exclusion criteria were as follows: presence of dual pathology (*n* = 0), intelligence quotient (IQ) < 70 (*n* = 1), and left‐handedness (*n* = 1). All procedures were part of the standard presurgical workup, and the study was approved by our local ethics board (CEP N^o^ 1129/2022). A written informed consent form, approved by our ethics committee, was obtained from all patients enrolled in this study.

### 2.2 Memory assessment

The presurgical neuropsychological evaluation comprised several tests to evaluate attention, executive functions, language, and memory, among others.^18^ Patients were divided based on side of the hippocampal resection. Verbal memory was evaluated with the Logical Memory (LM) test of the Wechsler Memory Scale Revised and Rey Auditory Verbal Learning Test (RAVLT). The normalization of test scores to *z*‐score was done based on mean and SD performances of an age‐ and sex‐matched control population provided in the test normalization tables. As employed elsewhere, raw data from LM was corrected by age and RAVLT by age and sex. For RAVLT, we used the performance in the last (fifth) learning trial as the learning score.

Based on long‐term (delayed recall) verbal memory scores of LM and RAVLT, patients were categorized into two groups: Deficit (*n* = 6), when delayed recall performance was ≤−1 on both tests or ≤−2 in one; and Preserved (*n* = 15), consisting of patients whose delayed recall memory scores were >−1 on at least one test and never ≤−2.

### 2.3 Immunohistochemistry

Formalin‐fixed paraffin‐embedded hippocampal sections at the body level were selected, and 3‐μm‐thick samples were immunostained to evaluate neuron density and IGF‐1R expression. Antibodies used were mouse anti‐NeuN (MAB377, Merck) and rabbit anti‐ IGF‐1Rβ (sc‐713, Santa Cruz Biotechnology), diluted at 1:4000 and 1:200. All immunostainings were performed with an EnVision FLEX, High pH (Link) kit (K8000, Dako) in an Autostainer Link 48 (Dako), following standard staining protocol. We performed counterstaining with hematoxylin.

Neuronal count was performed in NeuN‐stained sections for all hippocampal subfields, and density was estimated with Abercrombie's technique. To analyze IGF‐1R expression, we employed the threshold technique, using the *moments* algorithm to define the threshold. With this algorithm, the threshold is mathematically defined in each image based on average, SD, skewness, and kurtosis of the pixel intensity distribution. We measured the staining intensity and the immunopositive area (after removal of the counterstaining) and calculated the integrated optical density (IOD) based on both measurements. The IOD was chosen for the comparison because it resembles the measuring used in Western blot and factors both the area and staining intensity.

### 2.4 Statistical analysis

All statistics were performed with RStudio (version 2024.04.2+764) and R (version 4.4.2, 2024‐10‐31, “Pile of Leaves”). Numeric data were evaluated with Mann–Whitney, Crawford, or Student *t*‐test, based on data distribution and number, and categorical data were analyzed with Fisher test. The neuropsychological evaluations were correlated with histological data analysis and clinical variables using Pearson test.

## 3 RESULTS

### 3.1 Clinical data

The Preserved and Deficit groups were similar in all clinical data evaluated, including overall seizure frequency, percentage of patients with bilateral evolution, initial precipitant injury (IPI) occurrence and type, and postsurgical outcome. All clinical data are shown in Table 1.

**TABLE 1:** Clinical data from patients with preserved memory (Preserved group) and memory deficit (Deficit group).

Characteristic		Preserved	Deficit	*p*
Age at surgery, years		36 ± 7	40 ± 7	.36
Epilepsy onset, years		11 [1–28]	7 [6–25]	.79
IPI occurrence		47%	33%	.63
IPI type	Febrile seizure	57%	100%	.50
	Afebrile seizure	43%	0%	
Age at IPI, months		24 [3–36]	13 [2–24]	.56
Sex, female		80%	83%	1.00
Seizures per month		4 [1–12]	4 [1–30]	.96
Number of ASMs		2 [1–3]	2 [1–3]	.52
ASM dose, mg/day	CBZ	1200 [600–1600]	1000 [800–1600]	.81
	CLB	20 [10–30]	20 [10–40]	.75
	PB	50 [10–100]	20	.25^a^
	PHT	300 [200–400]	–	n.t.
	LMT	500 [200–800]	–	n.t.
	DZP	9	–	n.t.
	CLZ	2	–	n.t.
	VPA	–	1000	n.t.
ASMs taken	CBZ	80%	100%	.53
	CLB	60%	50%	1.00
	PB	20%	17%	1.00
	PHT	20%	0%	.53
	LMT	13%	0%	1.00
	DZP	7%	0%	1.00
	CLZ	7%	0%	1.00
	VPA	0%	17%	.29
Focal to bilateral evolution		40%	67%	.23
Psychiatric comorbidity		53%	50%	.63
Surgery type	TLR	33%	50%	.23
	SAH	67%	50%	
Engel IA outcome		33%	50%	.23

*Note*: Data are shown as average ± SD for parametric, median [minimum–maximum] for nonparametric, and percentage for categorical data.

Abbreviations: ASM, antiseizure medication; CBZ, carbamazepine; CLB, clobazam; CLZ, clonazepam; DZP, diazepam; IPI, initial precipitant injury; LMT, lamotrigine; n.t., not statistically tested; PB, phenobarbital; PHT, phenytoin; SAH, selective amygdalohippocampectomy; TLR, temporal lobe resection; VPA, valproic acid.

^a^ Crawford test was employed to compare PB, as only a single individual in the Deficit group took this medication.

### 3.2 Neuropsychological evaluation showed differences circumscribed to long‐term verbal memory in HS type 2

**FIGURE 1:**
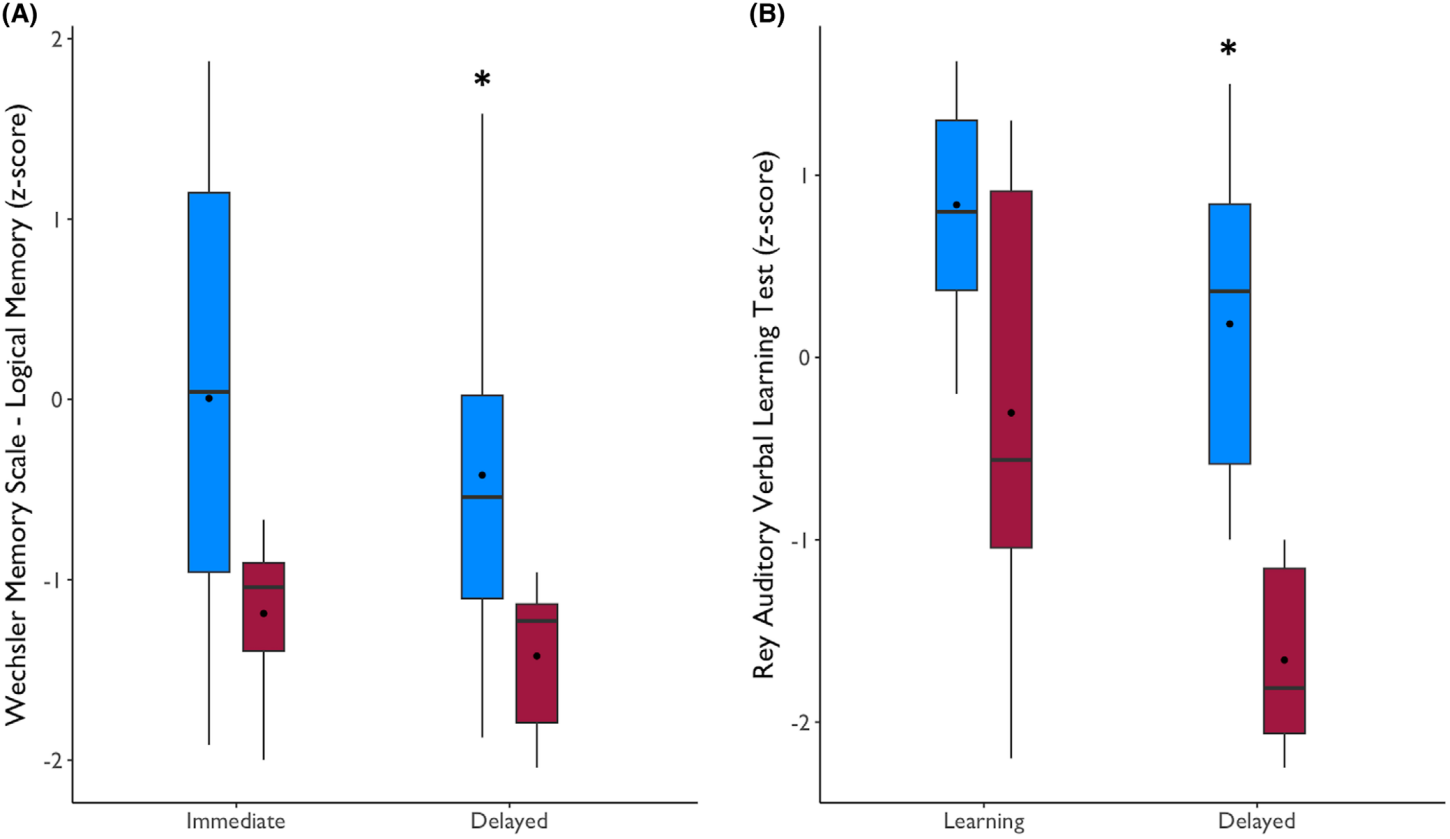
Performance of patients from the Preserved (blue boxplots) and Deficit (red boxplots) groups on the Logical Memory items of the Wechsler Memory Scale (A) and Rey Auditory Verbal Learning Test (B). As expected, the groups were statistically different in both late subitems, as these were used to categorize the patients into the groups. They were similar on immediate recall in Logical Memory and on learning in Rey's test. * *p* < .05.

Patients in both groups had similar education background (*p* = .14) and general IQ (*p* = .51). Verbal memory tests revealed lower performance in long‐term memory (delayed recall) on the Wechsler Memory Scale (*p* = .005; Figure 1A) and on delayed recall in RAVLT (*p* < .001; Figure 1B) in the Deficit group compared to the Preserved group. Immediate recall on the Wechsler Memory Scale and learning on RAVLT were similar between groups (*p* ≥ .05). A detailed presentation of neuropsychological data is provided in Table S1.

### 3.3 Hippocampal IGF‐1R expression correlates with long‐term verbal memory in HS type 2

**FIGURE 2:**
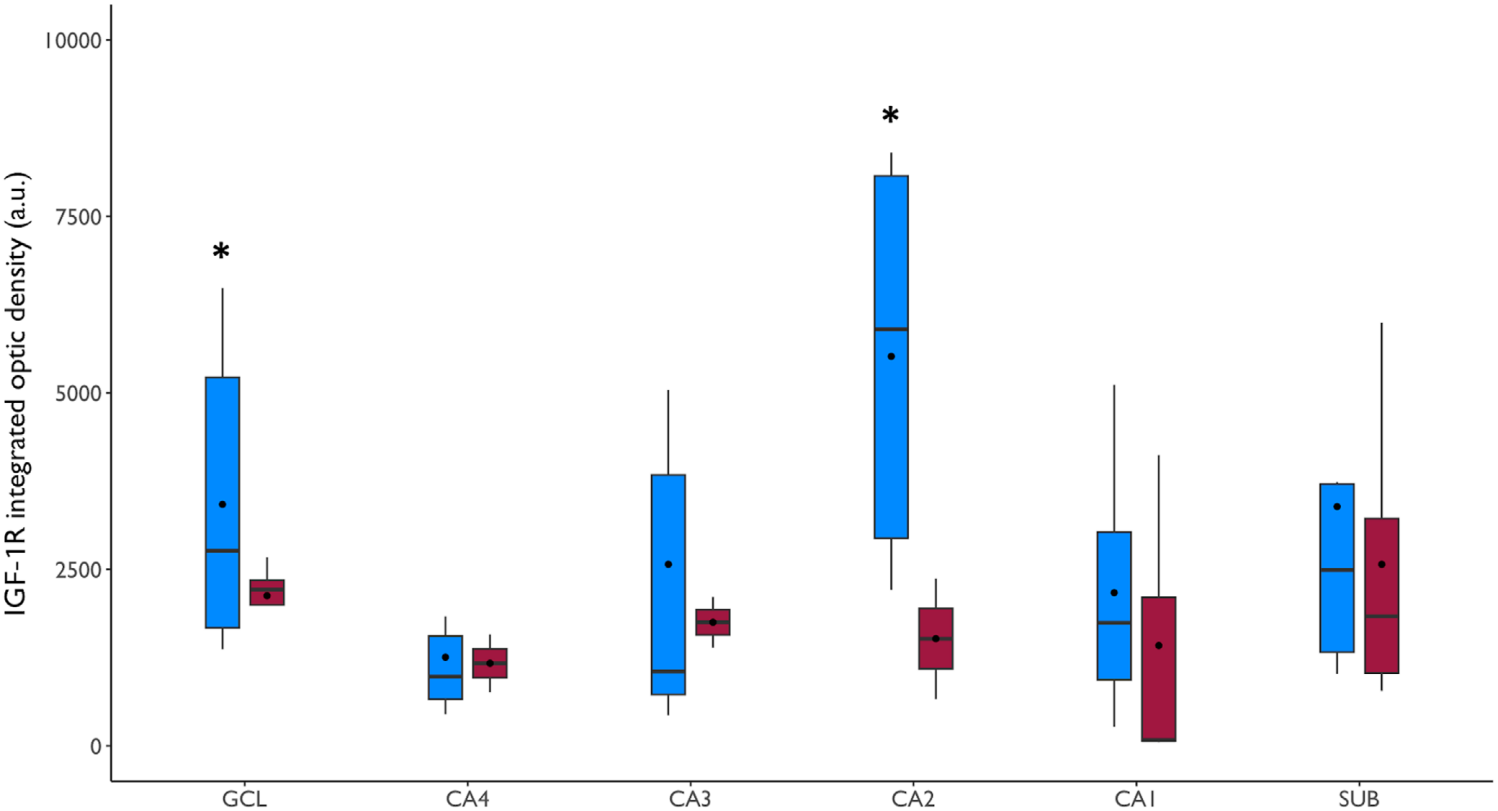
Insulinlike growth factor 1 receptor (IGF‐1R) integrated optic density (IOD) in the hippocampal subfields of patients from the Preserved (blue boxplots) and Deficit (red boxplot) groups. Patients from the Preserved group had higher IGF‐1R IOD in the granule cell layer (GCL) and cornu ammonis 2 (CA2). SUB, subiculum. * *p* < .05.

**FIGURE 3:**
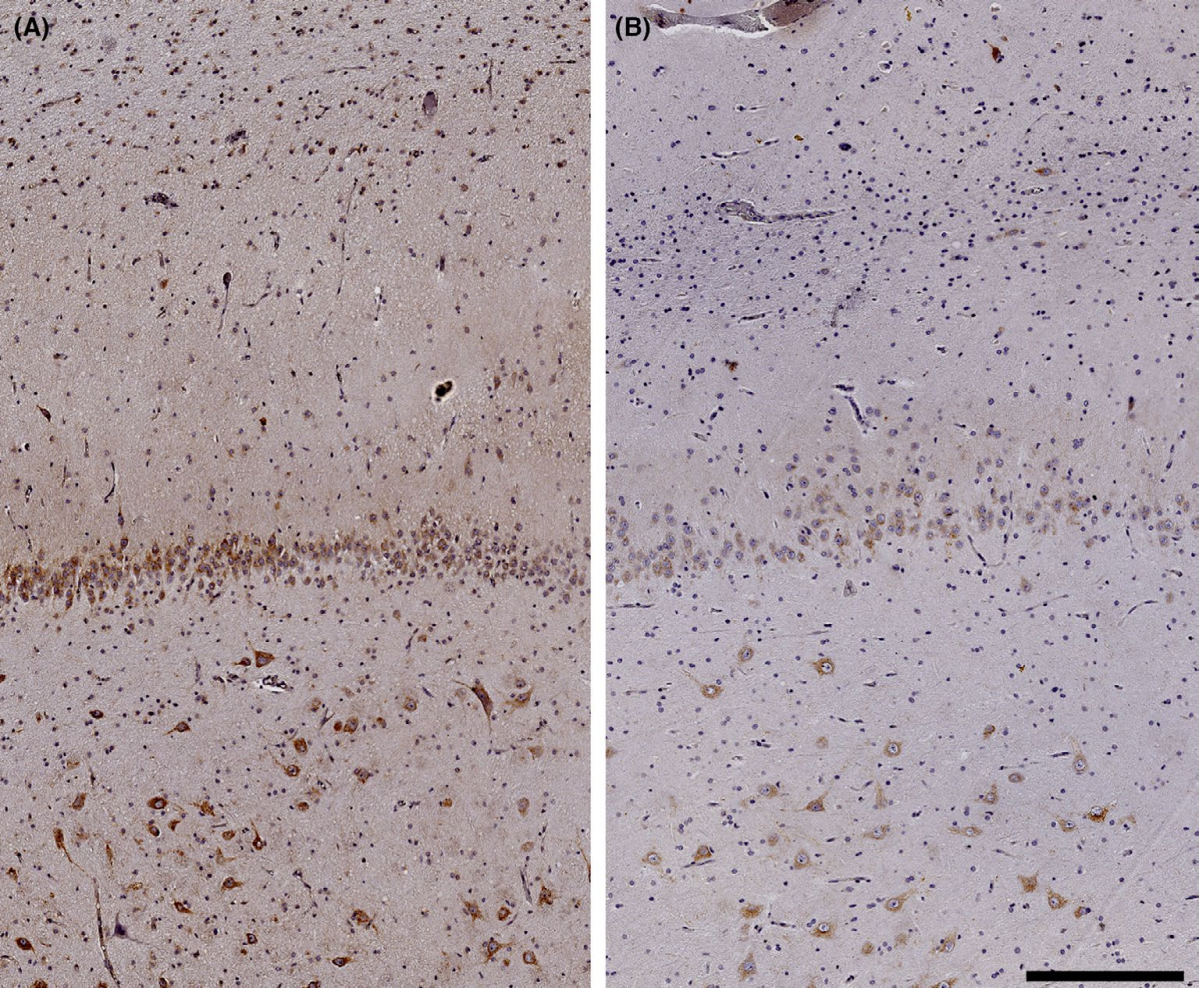
Representative images for insulin‐like growth factor 1 receptor immunohistochemistry in the external limb of the granule cell layer of a patient from the Preserved group (A) and from the Deficit group (B). See the noticeable neuropile staining in the Preserved group compared to the Deficit group. Scale bar = 250 μm.

**FIGURE 4:**
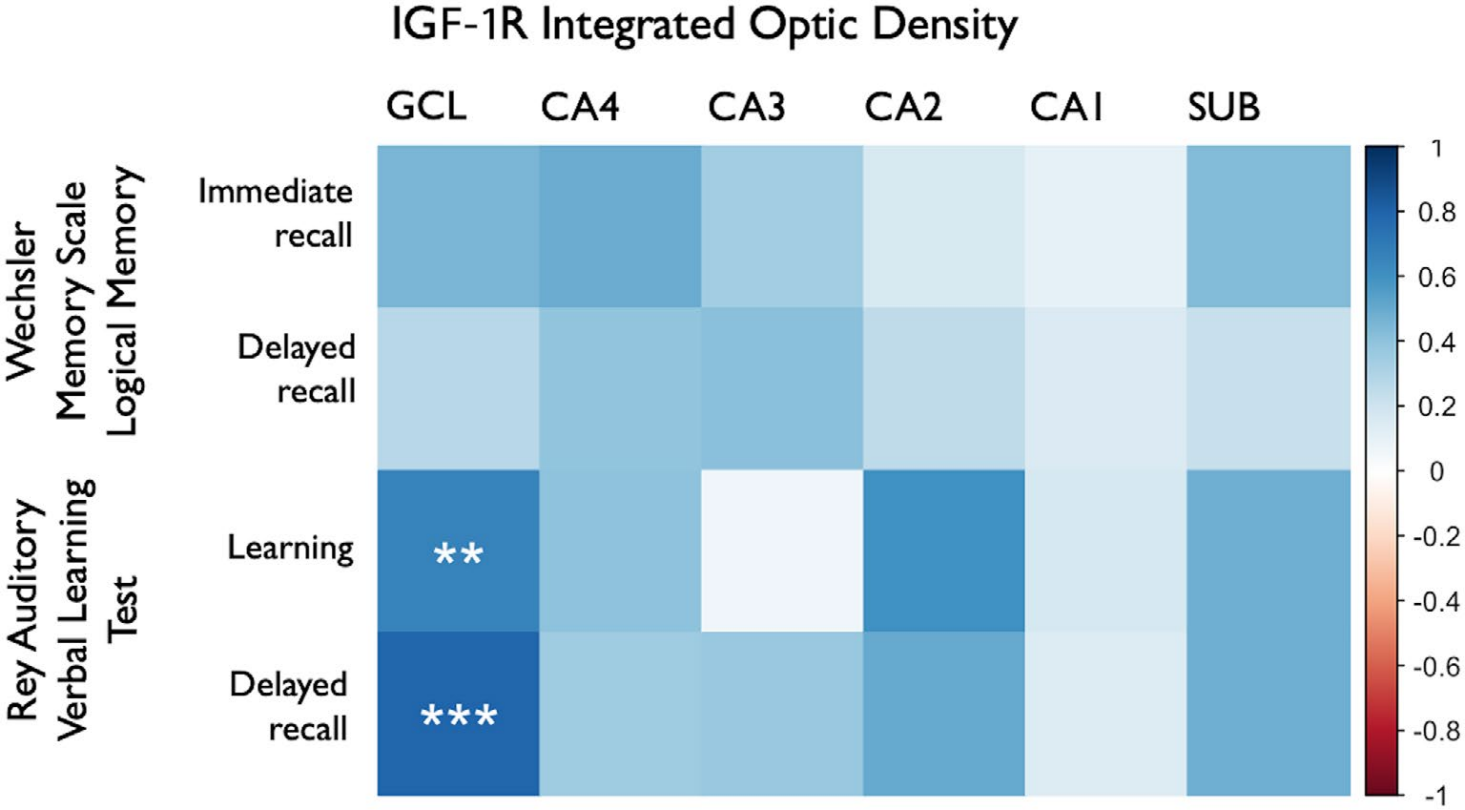
Correlation plot between insulin‐like growth factor 1 receptor (IGF‐1R) expression and neuropsychological data. The expression of IGF‐1R in the granule cell layer (GCL) had a strong positive correlation with learning and delayed recall scores on the Rey Auditory Verbal Learning Test. CA, cornu ammonis; SUB, subiculum. Statistical difference: **p < .01, ***p < .001.

The expression of IGF‐1R was higher in the GCL (*p* = .03) and CA2 (*p* = .02) of the Preserved group compared to the Deficit group (Figure 2). Both groups had similar IGF‐1R expression in CA4, CA3, CA1, and SUB (*p* ≥ .10). Moreover, whereas IGF‐1R immunostaining was seen in neuronal soma and in the neuropile of the molecular layer (indicative of dendritic staining) of patients from the Preserved group, cases from the Deficit group had only soma staining (Figure 3). IGF‐1R expression in the GCL correlated positively with immediate (*r* = .668, *p* = .01) and delayed recall (*r* = .793, *p* < .001) scores on RAVLT. IGF‐1R expression in the subiculum had a trend toward a positive correlation with the delayed recall subitem on RAVLT (*r* = .490, *p* = .05; Figure 4).

### 3.4 Neuron density was similar among patients with HS type 2 and different memory performances

**FIGURE 5:**
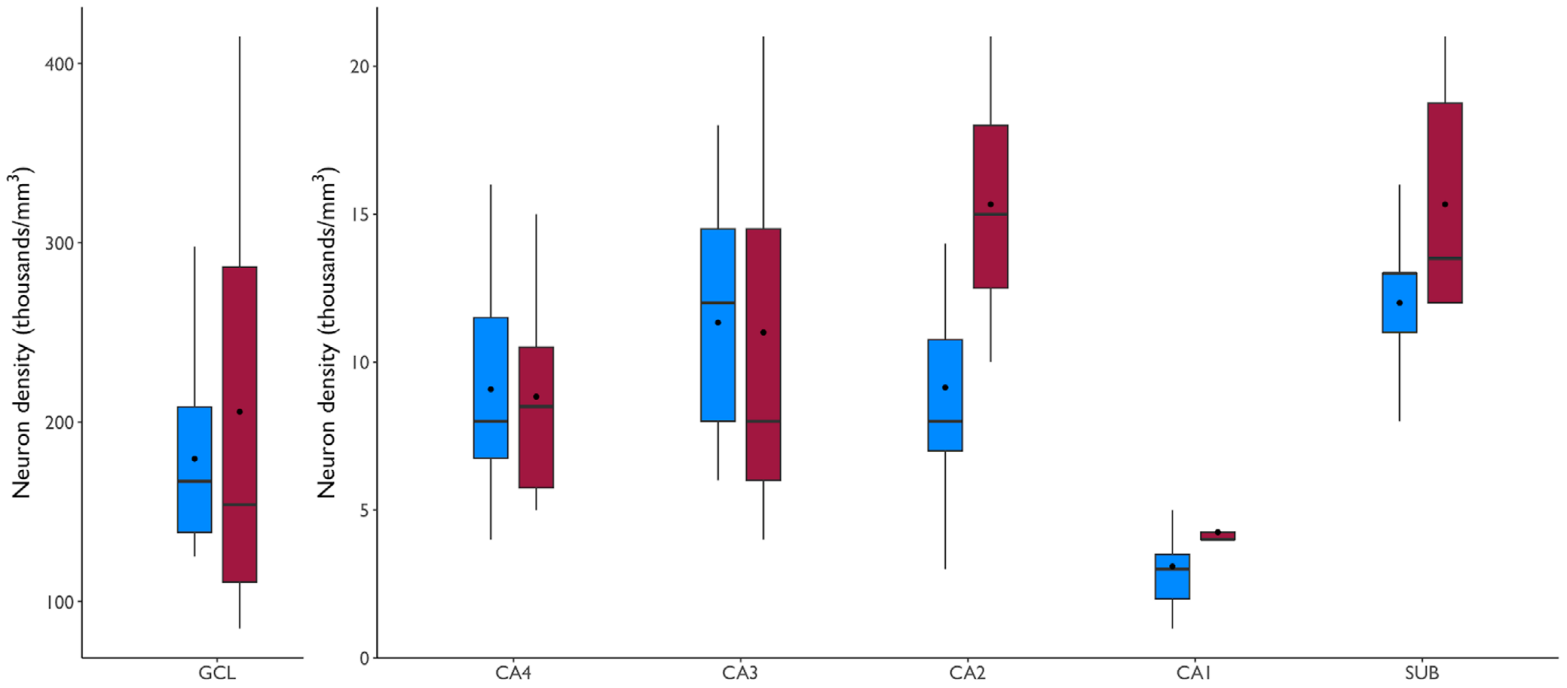
Neuron density in the hippocampal subfields of patients from the Preserved (blue boxplots) and Deficit (red boxplot) groups. Both groups had the same neuron density in all subfields. CA, cornu ammonis; GCL, granule cell layer; SUB, subiculum.

Patients from the Preserved group had the same neuron density as the Deficit group in the GCL, CA4, CA3, CA2, CA1, and SUB (*p* ≥ .45; Figure 5). Although neuron density in some regions had a higher average in the Deficit group, the median was similar or lower and the interquartile range was wider in this group. Neuron density did not correlate with LM immediate or delayed recall subitems or with RAVLT's immediate or delayed (*p* ≥ .08) recall subitems.

## 4 DISCUSSION

In the present study, we identified an association between long‐term verbal memory, measured with RAVLT, and IGF‐1R expression in patients with TLE and left HS type 2. Since the discovery of the crucial role of the hippocampal formation in long‐term declarative memory formation,^19^ several studies have investigated which changes in the hippocampus are related to mnemonic performance. Among patients with unilateral left‐side HS, most present with unilateral memory deficit, and those with normal memory scores have marked verbal memory decline after left temporal lobectomy.^18^ Another study showed a significant decline in verbal memory after left‐side temporal lobectomy in patients with severe HS, but no association between Wyler's HS severity classification and verbal memory scores.^20^ Most studies, however, associate hippocampal neuron density with memory performance.^21^, ^22^, ^23^ For instance, a study showed that patients with left TLE and severe neuronal loss had preoperative verbal memory deficits, whereas patients with moderate to mild loss had memory within expected ranges.^24^ Another showed a relationship of neuron density in CA4 and CA3 with verbal memory in patients with left TLE.^21^ Neuron density in the GCL has a strong correlation with memory performance in TLE patients with HS.^8^ Corroborating the importance of the GCL for memory, subsequent studies showed a strong correlation of granule cell density, granule neuron proliferation, and granule cell dendritic spine density with memory performance.^6^, ^10^, ^11^ In animal models, neonatal infection with Borna virus is associated with a reduced granule cell density and deficits in long‐term spatial memory, and neuron density is associated with memory performance in the pilocarpine model.^25^, ^26^, ^27^ Thus, the literature reinforces the role of hippocampal granule neurons in the mnemonic performance of patients with TLE.

Following the recent ILAE HS consensus classification, Coras et al. showed for the first time that patients with left‐side TLE and HS type 2 have preserved verbal memory, whereas those with HS type 1 and 3 present with verbal memory deficits.^6^ In a previous publication, we also saw a higher percentage of HS type 2 among patients with preserved memory.^7^ However, among the 13 patients with HS type 2, three (23%) had long‐term memory deficits.^6^ In the present study, we selected cases based only on having HS type 2 and surgery from the left side, without looking at neuropsychological scores, and found a similar proportion of HS type 2 patients with long‐term verbal memory deficit (29%). Because patients with type 2 sclerosis may present preservation to mild loss in the granular layer, and granule cells are strongly correlated with long‐term memory, we investigated whether variations in neuronal density in this region were associated with the mnemonic profile. We found no significant correlation between neuron density in any hippocampal subfield and memory performance. Although in several subfields the group with memory deficit had a higher average neuron density than the Preserved group, the differences were not significant. This apparent contradiction is caused by the higher interquartile range in all regions combined with the low number of cases of the Deficit group. For instance, the Deficit group presented with a 2.5× higher interquartile range in GCL and a 3.4× higher range in the subiculum than the Preserved group. The study of HS type 2 by Coras et al. showed a strong correlation between granule cell density and long‐term memory; however, their correlation evaluation included 78 patients with all HS types and also those without HS, and no correlation test was reported for HS type 2 alone, possibly due to the low number of cases.^6^ Overall, the variation in neuronal density in the granular layer and other regions of the patients with HS type 2 in our study may have caused the lack of association between neuron density and memory performance. Nevertheless, studies with a higher number of HS type 2 cases than ours should clarify this matter, especially given the stronger evidence for neuron density and memory performance from several studies.^6^, ^8^, ^10^

In our study, patients with HS type 2 and better memory scores had higher levels of IGF‐1R in the granular layer than those with verbal memory deficits. Changes in IGF‐1 were described in epilepsies of different etiologies. Among children with symptomatic infantile spasms, IGF‐1 is reduced in the cerebrospinal fluid, and this reduction was associated with poor response to treatment.^28^ Reduced serum IGF‐1 was also seen in the interictal period of both temporal and extratemporal epilepsy patients.^29^ This decrease was especially linked to TLE, longer epilepsy duration, and higher seizure frequency. In the brain tissue, however, a study showed that TLE patients had twice the IGF‐1 mRNA level, 50% higher IGF‐1 protein expression, and almost three times the phosphorylated IGF‐1R expression compared to control cases.^30^ Also, infusion of IGF‐1 prior to pilocarpine enhances excitability.^30^ On the other hand, there was no difference in serum IGF‐1 levels between children with tuberous sclerosis or with cryptogenic epileptic spasms compared to age‐matched controls.^31^ A murine model of infantile spasms revealed a deficit of IGF‐1 in interneurons and that treatment with IGF‐1 abolished spasms.^32^ Several animal models also link IGF‐1 fluctuations to seizures, showing a decrease in this neurotrophic factor's binding sites after kainic acid‐induced seizures or amygdala kindling.^33^, ^34^ In our study, however, we saw no correlation between IGF‐1R expression and seizure frequency, or epilepsy duration prior to surgery, and no difference in IGF‐1R expression between patients who presented with focal to bilateral tonic–clonic seizures and those without (*p* ≥ .08; data not shown). The previously mentioned studies showed that IGF‐1 expression is changed in patients with epilepsy and indicated a complex effect of IGF‐1 function on seizures. Further studies should, for instance, explore the relationship between seizures and changes in the IGF‐1 pathway, not only investigating seizure frequency but also evaluating how the time between last seizure and tissue collection impacts IGF‐1 or IGF‐1R expression.

Several studies showed a relationship between the IGF‐1 pathway and memory. Among elderly mice, IGF‐1R overexpression was associated with improved cognitive performance, with reduced time to complete the Morris water maze task and errors in this task.^35^ Under physiological conditions, physical exercise increases the activity of IGF‐1/IGF‐1R, which leads to increased expression of synapse‐related proteins and promotes better performance on the Morris water maze.^36^ Increased IGF‐1/IGF‐1R but not brain‐derived neurotrophic factor (BDNF)/tropomyosin receptor kinase B was associated with increased neurogenesis and better cognitive profile in gerbils treated with rufinamide.^37^ Among patients with Parkinson disease, those with lower serum IGF‐1 levels perform worse on RAVLT, both in immediate and delayed memory, and there was a moderate correlation between the scores on these items and IGF‐1R expression.^38^ In the kainic acid status epilepticus model, both IGF‐1 and neurogenesis are reduced in the chronic period, and treatment with IGF‐1 prevents spatial learning deficits, reduces the severity of seizures, prevents granular cell dispersion, reduces gliosis, and induces hippocampal neurogenesis.^39^, ^40^ In the hippocampus, IGF‐1R is specially increased in granule cell precursors, but can be found at lower levels in mature granule neurons and in CA pyramidal neurons.^15^ Because the GCL is one of the few regions of the adult brain where neurogenesis occurs, one of the possible mechanisms for the better performance of some patients would be the IGF‐1‐induced formation and incorporation of new neurons in the granular layer. Reinforcing this view, the *IGF1* gene is upregulated during epileptogenesis and after spontaneous seizures in the pilocarpine model of epilepsy.^41^ This increase may be related to mitogen‐activated protein kinase (MAPK)‐dependent neurogenesis, as in this model the fluctuation in IGF‐1 expression followed neurogenesis and blocking IGF‐1 receptor with AG1024 prevented both neurogenesis and MAPK pathway activation.^42^ Thus, IGF‐1‐induced neurogenesis may be one of the reasons for better memory performance in some HS type 2 patients.

In our study, although we cannot rule out neurogenesis as a factor in better memory in our patients, given the lack of difference in total neuronal density among the patients with preservation of or deficit in verbal memory, it is possible that IGF‐1R effects on memory in these patients are less influenced by neurogenesis and more by other physiological processes. Besides neurogenesis, IGF‐1 may impact memory via synaptic formation and function, as shown by some studies.^14^, ^35^, ^43^ Among the postsynaptic effects of this pathway, overexpression of IGF‐1 is associated with increased activation of the Ca^2+^/calmodulin‐dependent protein kinase II and extracellular signal‐regulated kinase (CaMKII‐ERK) pathway, which in turn affects long‐term potentiation.^35^ In CDKL5 mutant mice, IGF‐1 treatment rescues spine density and PSD‐95 expression to CDKL wild‐type phenotype levels.^44^ Reduced IGF‐1R expression is associated with reduction in synapses in a traumatic stress model.^43^ Other important functions of the IGF‐1 pathway are in cellular and mitochondrial protection against oxidative damage, inflammation, and edema, as shown in IGF‐1‐deficient mice.^45^, ^46^ Finally, although IGF‐1 is important for memory, this pathway may also have deleterious functions. Although IGF‐1R expression in astrocytes seems crucial for neuroprotection, studies show that IGF‐1R knockout in neurons is neuroprotective in Alzheimer models.^47^, ^48^, ^49^ In epilepsy, it may also have deleterious effects, as IGF‐1 was shown to have proseizure activity and blocking IGF‐1R with AXL1717 to have antiseizure effects.^30^, ^50^ However, studies in a model of malformations of cortical development showed that IGF‐1 treatment reduces N‐methyl‐D‐aspartate‐induced spasms and rescues the expression of synaptic‐related proteins.^51^ The same was seen in the tetrodotoxin model of infantile spams.^52^, ^53^ In short, the relationship between IGF‐1 pathway and memory can be related to several effects in the hippocampal neuron population, from neurogenesis to synaptic function.

Our semiquantitative measurements of IGF‐1R were performed in hippocampal sections at the hippocampal body level, a choice based on both methodological and functional considerations. Methodologically, our approach follows the ILAE guideline for HS classification, ensuring comparability with previous studies.^4^ Functionally, it aligns with the well‐established longitudinal specialization of the hippocampus, in which the dorsal/posterior regions of the hippocampus are primarily involved in memory processes, whereas the ventral/anterior region is more related to emotional processing.^4^ These functional specializations are related to several differences along the hippocampal longitudinal axis, ranging from the proportion of each hippocampal subfield along its longitudinal axis to differential connectivity, and in gene expression.^54^, ^55^ Cellular composition in a single subfield is also heterogeneous along the hippocampus, as shown in a comparison of neuronal transcriptome between the ventral and dorsal CA1 and GCL from mice and humans.^56^ Although IGF‐1R was not reported as differentially expressed in the longitudinal axis of these regions, future studies should investigate in depth whether there is any heterogeneity in the IGF1 pathway along the hippocampal axis.

Another important factor that may affect IGF‐1R expression is antiseizure medication (ASM) regimen. Some studies indicated that ASMs such as lamotrigine, phenytoin, topiramate, and oxcarbazepine increase serum levels of neurotrophic factors such as BDNF.^57^, ^58^, ^59^ Carbamazepine and oxcarbazepine, but not valproic acid or levetiracetam, were shown to increase serum IGF‐1, and rufinamide increases hippocampal expression of IGF‐1R.^37^, ^60^, ^61^ Among the ASMs taken by most of our patients at the time of the surgery, we saw no difference in IGF‐1R expression when comparing patients undertaking carbamazepine with those not taking this ASM or comparing those taking clobazam with those not taking this medication (*p* ≥ .11). Moreover, we saw no correlation between IGF‐1R expression and the daily doses of carbamazepine (*p* ≥ .22) or clobazam (*p* ≥ .188), or between IGF‐1R expression and the number of ASMs taken (*p* ≥ .53). Although we could not associate IGF‐1R expression with ASM type or dose in our dataset, larger studies should investigate this question.

In clinical terms, we saw no difference between patients with and without memory deficits. However, we saw some visible disproportion of febrile seizure in those from the Deficit group who had an IPI, as well as an increased frequency of focal to bilateral tonic–clonic seizures and a higher proportion of patients with good seizure outcome and who underwent anterior temporal lobe resection (TLR). In addition, both groups also had differences in ASM regiment, all nonsignificant. At least in terms of surgery type, selective amygdalohippocampectomy (SAH) is indicated for patients with left‐side TLE and preserved memory, as this approach presents a better neuropsychological outcome.^62^ This explains the disbalance in frequency of SAH among our groups. As for surgical outcome, several factors could be implicated, including the type of surgery and extension of the resection. Among the patients undergoing anterior temporal lobectomy, 67% were Engel IA versus only 30% of those undergoing SAH. Whereas some centers indicate no difference in surgical outcome between SAH and TLR, others link TLR to better postsurgical seizure control.^62^, ^63^, ^64^ Another factor is incomplete resection of the seizure onset zone, due to SAH with incomplete removal of hippocampal body or even the hippocampus tail or other mesial regions also being implicated in seizure generation.^65^ Besides surgery type, febrile seizures were also linked to better surgical outcome in a series of 621 patients with TLE,^63^ but this may be due to a higher frequency of febrile seizures among patients with HS type 1.^5^ Overall, these nonsignificant differences might be related to the small sample size, which should be explored in further studies.

This study is the first to indicate that IGF‐1 pathway can be one of the factors related to memory performance in patients with HS type 2. However, our study has several limitations. One important limitation of the present study is the number of patients, when seen only in absolute terms. However, HS type 2 is a rare and relatively recent pathology, only recognized worldwide after the 2013 ILAE classification of HS.^4^ In the seminal study of the German Reference Center for Epilepsy that showed memory preservation in HS type 2, only 13 patients were described and, considering only left‐side TLE patients, the number drops to five.^6^ Other studies from the same group and from others also reported a low number of HS type 2 cases among their samples,^7^, ^66^, ^67^, ^68^ as expected for this rare type of HS.^4^ Another limitation relates to our evaluation of the IGF‐1 pathway by immunohistochemistry of IGF‐1R. Although investigating the levels of IGF‐1 would be desired, a study indicated direct correlation between the amount of IGF‐1 and IGF‐1R; thus we believe IGF‐1R is a good indicator of IGF‐1 pathway activity in the central nervous system.^43^ In our study, we present *p*‐values not adjusted for multiple comparisons, as we only performed a limited number of statistical tests, each derived from an hypothesis based on literature data. Although adjustment for multiple comparisons is an important and necessary step in situations with great risk of incurring type I errors, such as exploratory investigations without an a priori hypothesis in large datasets, comparisons of multiple groups, or studies with several endpoints,^69^, ^70^ it is neither used nor indicated in situations where type II error would be increased.^69^ However, we present the false discovery rate‐adjusted values in the supplementary data (Table S2), as others may prefer this approach. As mentioned above, several clinical aspects where imbalanced in our groups, and although none was statistically different, further studies with a larger series should evaluate differences in epilepsy‐related clinical aspects in memory performance and IGF‐1 pathway expression in HS type 2. Finally, although we have not investigated HS types 1 and 3, there is no reason to believe the link between memory and IGF‐1 would be restricted to HS type 2. Determining whether similar mechanisms operate in other HS types represents an important and relevant direction for future research.

## 5 CONCLUSIONS

IGF‐1 pathway is associated with verbal memory performance in patients with left TLE and HS type 2, and reduced IGF‐1R expression in some cases may be one of the reasons for poor memory performance, in line with studies on animal models. Although we recon this as one of several factors behind memory performance in these patients, IGF‐1 pathway seems a promising venue of investigation of the molecular correlates of memory in HS.

## AUTHOR CONTRIBUTIONS

**Henrique Cruz:** Conceptualization; data curation; formal analysis; investigation; writing—original draft preparation; writing—review & editing. **Amauri Batista de Oliveira‐Júnior:** Conceptualization; data curation; formal analysis; investigation; writing—original draft preparation; writing—review & editing. **André Fernando Garcia Cortez:** Data curation; formal analysis; investigation. **Ricardo Lutzky Saute:** Data curation; formal analysis; investigation. **Ricardo Silva Centeno:** Data curation; funding acquisition; writing—review & editing. **Joao Norberto Stávale:** Data curation; investigation; writing—review & editing. **Mirian Salvadori Bittar Guaranha:** Data curation; writing—review & editing. **Elza Marcia Targas Yacubian:** Data curation; investigation; writing—review & editing. **Esper Abrão Cavalheiro:** Conceptualization; funding acquisition; investigation; writing—review & editing. **Joao Pereira Leite:** Conceptualization; funding acquisition; investigation; writing—review & editing. **Jose Eduardo Peixoto‐Santos:** Conceptualization; data curation; formal analysis; funding acquisition; investigation; supervision; writing—original draft preparation; writing—review & editing.

## CONFLICT OF INTEREST STATEMENT

None of the authors has any conflict of interest to disclose. We confirm that we have read the Journal's position on issues involved in ethical publication and affirm that this report is consistent with those guidelines.

## Supporting information


TABLES S1–S2

## Data Availability

The data that support the findings of this study are available on request from the corresponding author. The data are not publicly available due to privacy or ethical restrictions.
