# Supplementary material for: Insulin‐like growth factor 1 receptor correlates with verbal memory in ILAE type 2 hippocampal sclerosis
Source: Epilepsia. 2026 Feb 5;67(5):2572–84. doi: 10.1002/epi.70138 (PMC13179656; doi:10.1002/epi.70138)
Supplement: Supplementary file 1 — TABLES S1–S2 [file EPI-67-2572-s001.docx]

**Supplementary Table 1.** Raw and z-transformed scores comparisons of neuropsychological tests between patients with HS type 2 with preserved memory and with memory deficit.

| **Memory Test** | **Subitem** | **Measure** | **Preserved** | **Deficit** | **95% Confidence Interval** | **Effect size** | **p** | **p^FDR^** |
| --- | --- | --- | --- | --- | --- | --- | --- | --- |
| Logic Memory | Immediate | z-score | 0.01±1.21 | -1.19±0.48 | 0.42 - 1.97 | 0.442 | 0.05 | 0.52 |
|  |  | Raw | 26.85±6.01 | 17.33±5.39 | n.t. | n.t. | n.t. |  |
|  | Delayed | z-score | -0.42±0.98 | -1.42±0.46 | 0.34 - 1.66 | 0.493 | 0.005 | 0.15 |
|  |  | Raw | 20.4±5.64 | 10.50±5.68 | n.t. | n.t. | n.t. |  |
| RAVLT | Learning | z-score | 0.84±0.57 | -0.30±1.39 | -0.32 - 2.60 | 0.425 | 0.10 | 0.52 |
|  |  | Raw | 13.92±1.19 | 11.83±2.79 | n.t. | n.t. | n.t. |  |
|  | Delayed | z-score | 0.18±0.85 | -1.66±0.55 | 1.17 - 2.52 | 0.734 | <0.001 | 0.03 |
|  |  | Raw | 11.69±2.53 | 6.83±1.72 | n.t. | n.t. | n.t. |  |

n.t. = not tested (raw scores, as they are unadjusted by control values, were not statistically tested).

**Supplementary Table 2.** Unadjusted and FDR-corrected p-values for all comparisons.

| Comparison | Data | p | p^FDR^ |
| --- | --- | --- | --- |
| Preserved vs Deficit | Age at surgery (years) | 0.36 | 0.68 |
| Preserved vs Deficit | Epilepsy onset (years) | 0.79 | 0.95 |
| Preserved vs Deficit | IPI occurrence | 0.63 | 0.82 |
| Preserved vs Deficit | IPI type | 0.50 | 0.80 |
| Preserved vs Deficit | Age at IPI (months) | 0.56 | 0.82 |
| Preserved vs Deficit | Sex (Female) | 1.00 | 1.00 |
| Preserved vs Deficit | Seizures per month | 0.96 | 1.00 |
| Preserved vs Deficit | Number of ASM | 0.52 | 0.80 |
| Preserved vs Deficit | CBZ dose | 0.81 | 0.96 |
| Preserved vs Deficit | CLB dose | 0.75 | 0.93 |
| Preserved vs Deficit | FB dose | 0.25 | 0.57 |
| Preserved vs Deficit | CBZ (%) | 0.53 | 0.80 |
| Preserved vs Deficit | CLB (%) | 1.00 | 1.00 |
| Preserved vs Deficit | FB (%) | 1.00 | 1.00 |
| Preserved vs Deficit | PHT (%) | 0.53 | 0.80 |
| Preserved vs Deficit | LMT (%) | 1.00 | 1.00 |
| Preserved vs Deficit | DZP (%) | 1.00 | 1.00 |
| Preserved vs Deficit | CLZ (%) | 1.00 | 1.00 |
| Preserved vs Deficit | VPA (%) | 0.29 | 0.61 |
| Preserved vs Deficit | Focal-to-bilateral evolution (%) | 0.23 | 0.57 |
| Preserved vs Deficit | Psychiatric comorbidity | 0.63 | 0.82 |
| Preserved vs Deficit | Years of education | 0.14 | 0.57 |
| Preserved vs Deficit | IQ | 0.51 | 0.80 |
| Preserved vs Deficit | Surgery type | 0.23 | 0.57 |
| Preserved vs Deficit | Engel IA | 0.23 | 0.57 |
| Preserved vs Deficit | LM Immediate | 0.05 | 0.52 |
| Preserved vs Deficit | LM Delayed | 0.01 | 0.15 |
| Preserved vs Deficit | RAVLT L | 0.10 | 0.52 |
| Preserved vs Deficit | RAVLT Delayed | 0.00 | 0.03 |
| Preserved vs Deficit | NeuN GCL | 0.67 | 0.86 |
| Preserved vs Deficit | NeuN CA4 | 0.45 | 0.76 |
| Preserved vs Deficit | NeuN CA3 | 0.47 | 0.77 |
| Preserved vs Deficit | NeuN CA2 | 0.91 | 1.00 |
| Preserved vs Deficit | NeuN CA1 | 0.95 | 1.00 |
| Preserved vs Deficit | NeuN SUB | 0.94 | 1.00 |
| Preserved vs Deficit | IGF GCL | 0.03 | 0.45 |
| Preserved vs Deficit | IGF CA4 | 0.44 | 0.76 |
| Preserved vs Deficit | IGF CA3 | 0.10 | 0.52 |
| Preserved vs Deficit | IGF CA2 | 0.02 | 0.36 |
| Preserved vs Deficit | IGF CA1 | 0.32 | 0.63 |
| Preserved vs Deficit | IGF SUB | 0.27 | 0.59 |
| Correlation | NeuN GCL vs LM Immediate | 0.16 | 0.57 |
| Correlation | NeuN GCL vs LM Delayed | 0.19 | 0.57 |
| Correlation | NeuN GCL vs RAVLT Learning | 0.20 | 0.57 |
| Correlation | NeuN GCL vs RAVLT Delayed | 0.38 | 0.70 |
| Correlation | NeuN CA4 vs LM Immediate | 0.20 | 0.57 |
| Correlation | NeuN CA4 vs LM Delayed | 0.62 | 0.82 |
| Correlation | NeuN CA4 vs RAVLT Learning | 0.78 | 0.95 |
| Correlation | NeuN CA4 vs RAVLT Delayed | 1.00 | 1.00 |
| Correlation | NeuN CA3 vs LM Immediate | 0.20 | 0.57 |
| Correlation | NeuN CA3 vs LM Delayed | 0.22 | 0.57 |
| Correlation | NeuN CA3 vs RAVLT Learning | 0.22 | 0.57 |
| Correlation | NeuN CA3 vs RAVLT Delayed | 0.98 | 1.00 |
| Correlation | NeuN CA2 vs LM Immediate | 0.88 | 1.00 |
| Correlation | NeuN CA2 vs LM Delayed | 0.59 | 0.82 |
| Correlation | NeuN CA2 vs RAVLT Learning | 0.12 | 0.53 |
| Correlation | NeuN CA2 vs RAVLT Delayed | 0.40 | 0.73 |
| Correlation | NeuN CA1 vs LM Immediate | 0.26 | 0.58 |
| Correlation | NeuN CA1 vs LM Delayed | 0.30 | 0.61 |
| Correlation | NeuN CA1 vs RAVLT Learning | 0.30 | 0.61 |
| Correlation | NeuN CA1 vs RAVLT Delayed | 0.71 | 0.89 |
| Correlation | NeuN SUB vs LM Immediate | 0.12 | 0.53 |
| Correlation | NeuN SUB vs LM Delayed | 0.09 | 0.52 |
| Correlation | NeuN SUB vs RAVLT Learning | 0.15 | 0.57 |
| Correlation | NeuN SUB vs RAVLT Delayed | 0.08 | 0.52 |
| Correlation | IGF GCL vs LM Immediate | 0.09 | 0.52 |
| Correlation | IGF GCL vs LM Delayed | 0.33 | 0.64 |
| Correlation | IGF GCL vs RAVLT Learning | 0.01 | 0.22 |
| Correlation | IGF GCL vs RAVLT Delayed | 0.00 | 0.03 |
| Correlation | IGF CA4 vs LM Immediate | 0.09 | 0.52 |
| Correlation | IGF CA4 vs LM Delayed | 0.18 | 0.57 |
| Correlation | IGF CA4 vs RAVLT Learning | 0.21 | 0.57 |
| Correlation | IGF CA4 vs RAVLT Delayed | 0.24 | 0.57 |
| Correlation | IGF CA3 vs LM Immediate | 0.25 | 0.57 |
| Correlation | IGF CA3 vs LM Delayed | 0.15 | 0.57 |
| Correlation | IGF CA3 vs RAVLT Learning | 0.84 | 0.98 |
| Correlation | IGF CA3 vs RAVLT Delayed | 0.21 | 0.57 |
| Correlation | IGF CA2 vs LM Immediate | 0.63 | 0.82 |
| Correlation | IGF CA2 vs LM Delayed | 0.45 | 0.76 |
| Correlation | IGF CA2 vs RAVLT Learning | 0.08 | 0.52 |
| Correlation | IGF CA2 vs RAVLT Delayed | 0.11 | 0.53 |
| Correlation | IGF CA1 vs LM Immediate | 0.71 | 0.89 |
| Correlation | IGF CA1 vs LM Delayed | 0.58 | 0.82 |
| Correlation | IGF CA1 vs RAVLT Learning | 0.58 | 0.82 |
| Correlation | IGF CA1 vs RAVLT Delayed | 0.62 | 0.82 |
| Correlation | IGF SUB vs LM Immediate | 0.10 | 0.52 |
| Correlation | IGF SUB vs LM Delayed | 0.41 | 0.73 |
| Correlation | IGF SUB vs RAVLT Learning | 0.08 | 0.52 |
| Correlation | IGF SUB vs RAVLT Delayed | 0.05 | 0.52 |
